# Adverse effects and non-adherence to antihypertensive medications in University of Gondar Comprehensive Specialized Hospital

**DOI:** 10.1186/s40885-018-0104-6

**Published:** 2019-01-15

**Authors:** Eyob Alemayehu Gebreyohannes, Akshaya Srikanth Bhagavathula, Tamrat Befekadu Abebe, Yonas Getaye Tefera, Tadesse Melaku Abegaz

**Affiliations:** School of Pharmacy, University of Gondar-College of Medicine and Health Sciences, Gondar, Ethiopia

**Keywords:** Hypertension, Antihypertensive medications, Adherence, Adverse effects, Gondar, Ethiopia

## Abstract

**Background:**

A considerable proportion of cardiovascular events could be attributed to poor adherence to antihypertensive medications. Adverse effects can be severe enough to affect adherence to antihypertensive medications. This study aimed to measure the contribution of adverse effects on antihypertensive medications adherence.

**Methods:**

The study was conducted from May 1 to June 30, 2017, at the ambulatory clinic of University of Gondar Comprehensive Specialized Hospital (UOGCSH) in Gondar town. A binary logistic regression was performed to determine the significance of the association between adverse effects and adherence to antihypertensive medications. An institution-based cross-sectional study was conducted by administering a questionnaire to hypertensive patients who came for follow-up at the ambulatory clinic of UOGCSH. Level of adherence to antihypertensive medications was used as outcome measure.

**Results:**

A total of 249 patients were included in the final analysis with a mean age of 56.51 years and a female majority (53%). The following variables were identified as predictors of poor adherence: tiredness [AOR (95% CI): 3.802 (1.723–8.391), *p* = 0.001], muscle pain [AOR (95% CI): 5.199 (1.407–19.214), *p* = 0.013], poor sleep [AOR (95% CI): 4.891 (1.578–15.160), *p* = 0.006] and, believing that the symptoms were caused by antihypertensive medications [AOR (95% CI): 3.249 (1.248–8.456), *p* = 0.016].

**Conclusion:**

Adverse effect significantly contributes to antihypertensive medication non-adherence among hypertensive patients.

## Background

The most important goal of antihypertensive drug treatment is to reduce renal and cardiovascular morbidity and mortality by lowering blood pressure (BP). To achieve this goal patients should be encouraged to adhere to the prescribed pharmacologic and non-pharmacologic management strategies [1]. A considerable proportion of cardiovascular events such as angina, myocardial infarction, chronic heart failure, kidney failure, transient cerebral ischemic attacks and strokes, premature mortality and disability, and increased cost of hospitalization could be attributed to poor adherence to antihypertensive medications and persistently elevated BP [2–5]. On the other hand, significantly lower systolic and diastolic BPs have been achieved by hypertensive patients reporting strict adherence to prescribed drug therapy than those who admit even an occasional lapse in taking their antihypertensive medications [6, 7]. In addition, an increased level of adherence to antihypertensive agents (≥80%) provides a net economic gain by decreasing the occurrence of hypertension-associated complications [4, 5].

Patients report intentional and unintentional non-adherence. While unintentional nonadherence is a passive process whereby patients may be unconcerned or forgetful about adhering to their antihypertensive medications, intentional nonadherence, on the other hand, can be considered an active process whereby patients deliberately stray from adhering to their antihypertensive medication treatment [8]. Even though life-threatening adverse effects and hospitalization for adverse effects are rare occurrences, adverse effects such as cough, edema, flush, headache, increased urination, rapid pulse, wheezing/shortness of breath and dizziness can be severe enough to affect the utilization of antihypertensive medications [8–20]. The numbers of adverse effects reported by individual patients are also of paramount importance in patients’ adherence to their antihypertensive medications [8].

A comparison of the answers of the patients and physicians on medication adherence showed that adherence may be overestimated by the doctors. This indicates that patients are usually less adherent to the prescribed medications than what their doctors’ think [14]. Therefore, a properly formulated questionnaire can help distinguish adherent patients from those who are non-adherent [21].

Adherence to antihypertensive medications is a concern in the University of Gondar Comprehensive Specialized Hospital (UOGCSH) as evidenced by the low level of adherence. One-third (35.4%) of hypertensive patients attending UOGCSH were found to be non-adherent to their prescribed anti-hypertensive treatment [22]. However, the contribution of adverse events for the low level of adherence remains unknown. Therefore, the aim of this study was to measure the contribution of adverse effects on antihypertensive medications adherence.

## Methods

### Study setting and period

The study was conducted from May 1 to June 30, 2017, at the ambulatory clinic of UOGCSH in Gondar town. Gondar is a historical town located in the northwest part of Ethiopia, 725 km from the capital city Addis Ababa.

### Study design

An institution-based cross-sectional study was conducted by administering a questionnaire to hypertensive patients who came for follow-up at the ambulatory clinic of UOGCSH.

### Data collection procedures

The data collection tool was adopted from the Duarte-Silva et al. study [23]. In addition, since 80% has been used by several studies as a cut-off point for good adherence, the questionnaire has been modified to include this [24–27]. Before the data collection, the questionnaire was first translated into Amharic by one of the investigators (EAG) and then back-translated into English by the other investigator (TBA) for verifying accuracy. Discrepancies were dealt with a discussion. The questionnaire had been pretested on 25 hypertensive patients before it was administered to check if the patients were able to understand it. These patients were excluded from the final analyses and no modification was made on the questionnaire after the pretest.

The questionnaire was basically self-administered; however, for study participants who faced difficulty or who were illiterates, an interview was used as a means of data collection. Data on patients’ current antihypertensive medication regimens were taken from their medical records.

Level of adherence was calculated by subtracting the number of missed doses (obtained from the questionnaire) from the total intended doses (obtained from medical records) and calculating the adherence out of 100%. There were questions on the questionnaire stating: “*Many patients have difficulties taking their medicines as the doctor recommended. In the past three months, there was any day or period of time when you did not take the drugs for blood pressure as recommended?”* and “*In a month, how many times did that happen?*”

### Inclusion and exclusion criteria

All hypertensive patients aged 18 years and above who came to the ambulatory clinic of UOGCSH and were taking one or more antihypertensive medications for the management of their high BP at least for 3 months were included in the study. The following patients were excluded from the study: patients who do not consent to participate; patients who didn’t completely fill out the questionnaire.

Level of adherence to antihypertensive medications was used as an outcome measure. Sociodemographic characteristics, the antihypertensive medications used, perceived adverse effects by patients, whether patients spoke about adverse effects, whether patients think that perceived symptoms were caused by antihypertensive medications, and the non-pharmacologic management strategies taken were used as independent variables.

### Statistical analysis

Descriptive statistics were used to present patient characteristics. Shapiro – Wilk and Levene’s tests were performed to assess the data for normality and homogeneity. The four-item Likert scale was dichotomized into two: Never/Sometimes and Usually/Always. A binary logistic regression was performed to determine the significance of the association between adverse effects and adherence to antihypertensive medications. Variables with *p* ≤ 0.20 in univariate were included to perform multivariate analysis. Similarly, significance of association between specific antihypertensive medications and adverse effects were determined using binary logistic regression analysis. All data analyses were done using the statistical package for social sciences (SPSS 20).

### Ethics approval

Ethical clearance was obtained from the research ethical review committee of the school of Pharmacy, University of Gondar. Written informed consent was sought from each study participant and confidentiality of the information was assured in such a way that no disclosure of any information obtained from the participants in relation to the finding was made and the information was used for the research purpose only.

## Results

A total of 249 patients were included in the final analysis with a mean age of 56.51 years and a female majority (53%). Most of the study participants lived in urban areas and were married. The number of antihypertensive medications used by patients ranges from one to three. Nearly one-third of the study participants also used additional medications for the management of other chronic conditions (Table 1).

**Table 1:** Baseline characteristics of hypertensive patients attending UOGCSH (*N* = 249)

Characteristics	
Age	
Mean (±standard deviation)	56.51 (11.004)
Age at time of HTN diagnosis	
Median (interquartile range)	50 (11)
Duration of antihypertensive medication use (years)	
Median (interquartile range)	4 (5)
Sex N (%)	
Female	132 (53%)
Residence N (%)	
Urban	164 (65.9%)
Marital status N (%)	
Single	22 (8.8%)
Married	198 (79.5%)
Divorced	12 (4.8%)
Widowed	17 (6.8%)
Education level N (%)	
No formal education	99 (39.8%)
Primary education	67 (26.9%)
Secondary education	32 (12.9%)
College and above	51 (20.5%)
Smoking status N (%)	
Yes	13 (5.2%)
Number of antihypertensive medications used	
Median (Range)	2 (1–4)
Other medication use N (%)	86 (34.5%)

**Fig. 1:**
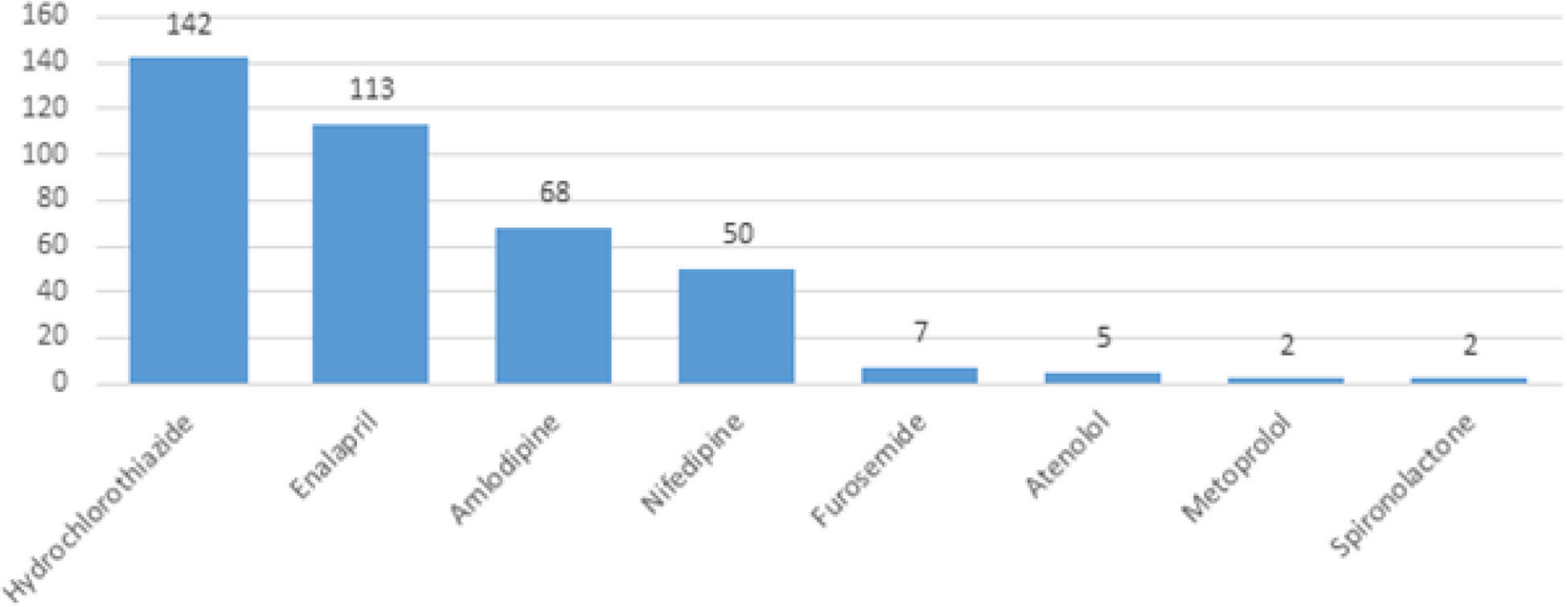
The number of hypertensive patients using different antihypertensive medications at UOGCSH (*N* = 249)

Hydrochlorothiazide (HCT) was the most commonly prescribed -medication, followed by enalapril and amlodipine (Fig. 1). The median (minimum-maximum) daily doses of HCT, enalapril, amlodipine, and nifedipine were 25 mg (12.5–50), 5 mg (2.5–20), 5 mg [5–20], and 20 mg (5–40), respectively.

Nearly one-third of patients had poor adherence (< 80%) to their antihypertensive medications. Forgetfulness (20.1%) and fasting (5.6%) were the two most commonly mentioned reasons for poor adherence (Table 2). Approximately 60% of the study participants didn’t know if symptoms were caused by their antihypertensive medications. Among the study participants, 21.3% think that their symptoms were caused by their antihypertensive medications while 13.4% of the study participants reported that, symptoms caused them to change their medication administration behavior (Additional file 1).

**Table 2:** Adherence to antihypertensive medications among hypertensive patients attending UOGCSH (*N* = 249)

	Number (Percent)
Level of adherence	
Good (≥80%)	156 (62.7%)
Poor (< 80%)	93 (37.3%)
Reasons for poor adherence	
Forgetfulness	50 (20.1%)
Economic reasons	6 (2.4%)
Drug cause discomfort or malaise	6 (2.4%)
BP was normal	1 (0.4%)
BP was too low	3 (1.2%)
Unavailability of drugs in the market	7 (2.8%)
Fasting	14 (5.6%)
Traveling	5 (2.0%)
Forgetfulness and economic reasons	1 (0.4%)
Importance of missed dose on BP	
None	160 (64.3%)
Little	31 (12.4%)
Some	25 (10.0%)
Many	33 (13.3%)

Among the study participants, 43.37% were taking monotherapy while the remaining patients were on two or more combination antihypertensive medications. Thiazides, Thiazide/Angiotensin Converting Enzyme Inhibitors (ACEIs), and Thiazide/ACEIs/Calcium Channel Blockers (CCBs) were the most commonly used single, two-drug, and three-drug therapies, respectively (Additional file 2). Twenty-five different types of adverse effects were reported by the study participants. Tiredness and dizziness were the two most commonly occurring adverse effects being reported by nearly half of the study participants as “Usually/Always” (Table 3).

**Table 3:** Adverse effects experienced by hypertensive patients attending UOGCSH (*N* = 249)

Adverse effects	Good adherence (*N* = 156)		Poor adherence (*N* = 93)		Total (*N* = 249)	
	Never/ Sometimes N (%)	Usually/ Always N (%)	Never/ Sometimes N (%)	Usually/ Always N (%)	Never/ Sometimes N (%)	Usually/ Always N (%)
Tiredness	98 (62.8)	58 (37.2)	32 (34.4)	61 (65.6)	130 (52.2)	119 (47.8)
Sweats	138 (88.5)	18 (11.5)	73 (78.5)	20 (81.5)	211 (84.7)	38 (15.3)
Gripes	142 (91.0)	14 (9.0)	80 (86.0)	13 (14.0)	222 (89.2)	27 (10.8)
Nausea	140 (89.7)	16 (10.3)	77 (82.8)	16 (17.2)	217 (87.1)	32 (12.9)
Diarrhea	152 (97.4)	4 (2.6)	92 (98.9)	1 (1.1)	244 (88.0)	5 (2.0)
Constipation	131 (84.0)	25 (16.0)	82 (88.2)	11 (11.8)	212 (85.5)	36 (14.5)
Palpitations	114 (73.1)	42 (26.9)	53 (57.0)	40 (43.0)	167 (67.1)	82 (32.9)
Swollen feet or legs	120 (76.9)	36 (23.1)	67 (72.0)	26 (28.0)	187 (75.1)	62 (24.9)
Cold hands or feet	145 (92.9)	11 (7.1)	75 (80.6)	18 (19.4)	220 (88.4)	29 (11.6)
Muscle pain	146 (93.6)	10 (6.4)	77 (82.8)	16 (17.2)	223 (89.6)	26 (10.4)
Cramps	146 (93.6)	10 (6.4)	82 (88.2)	11 (11.8)	228 (91.6)	21 (8.4)
Headaches	91 (58.3)	65 (41.7)	45 (48.4)	48 (51.6)	136 (54.6)	113 (45.4)
Dizziness	83 (53.2)	73 (46.8)	47 (50.5)	46 (49.5)	130 (52.2)	119 (47.8)
Anxiety	142 (91.0)	14 (9.0)	78 (83.9)	15 (16.1)	220 (88.4)	29 (11.6)
Sadness	143 (91.7)	13 (8.3)	81 (87.1)	12 (12.9)	224 (90)	25 (10.0)
Sleep poorly	136 (87.2)	20 (12.8)	71 (76.3)	22 (23.7)	207 (83.1)	42 (16.9)
Shortness of breath or breathing difficulty	142 (91.0)	14 (9.0)	84 (90.3)	9 (9.7)	226 (90.8)	23 (9.2)
Persistent dry cough	124 (79.5)	32 (20.5)	70 (75.3)	23 (24.7)	194 (77.9)	55 (22.1)
Itching	146 (93.6)	10 (6.4)	87 (93.5)	6 (6.5)	233 (93.6)	16 (6.4)
Skin rash	151 (96.8)	5 (3.2)	84 (90.3)	9 (9.7)	235 (94.4)	14 (5.6)
Swollen or red face	150 (96.2)	6 (3.8)	92 (98.9)	1 (1.1)	242 (97.2)	7 (2.8)
Dry mouth	142 (91.0)	14 (9.0)	82 (88.2)	11 (11.8)	224 (90)	25 (10.0)
Frequent urination	98 (62.8)	58 (37.2)	61 (65.6)	32 (34.4)	159 ()	90 (36.1)
Decreased sexual desire or ability sexual	126 (80.8)	30 (19.2)	83 (89.2)	10 (10.8)	208 ()	40 (16.1)
Blurred vision	155 (99.4)	1 (0.6)	93 (100.0)	0 (0.0)	248 ()	1 (0.4)

Univariate analysis of binary logistic regression identified age, duration of antihypertensive medication use, and use of nifedipine and medications other than those used for management of hypertension as predictors of poor adherence. However, none of these were able to independently predict poor adherence to antihypertensive medications up on multivariate analysis. Multivariate analysis identified that patients who selected “Usually/Always” for tiredness [adjusted odds ratio (AOR) (95% confidence interval (CI)): 3.802 (1.723–8.391), *p* = 0.001], muscle pain [AOR (95% CI): 5.199 (1.407–19.214), *p* = 0.013], and poor sleep [AOR (95% CI): 4.891 (1.578–15.160), *p* = 0.006] were more likely to have poor adherence to their antihypertensive medication doses. In addition, patients who believe that the symptoms they experienced were caused by their antihypertensive medications were more likely to have poor adherence than patients who do not know [AOR (95% CI): 3.249 (1.248–8.456), *p* = 0.016]. More importantly, patients who reported that symptoms caused them to change their antihypertensive medication-taking behavior were more likely to have poor adherence to their antihypertensive medication doses [AOR (95% CI): 16.104 (4.164–62.290), *p* = 0.000]. On the other hand, patients who reported having regular physical exercise were more likely to have good adherence [AOR (95% CI): 0.170 (0.072–0.402), *p* = 0.000] (Table 4).

**Table 4:** Predictors of poor adherence to antihypertensive medications among hypertensive patients attending UOGCSH (*N* = 249)

Variables	Crude odds ratio (COR) (95% CI)	*p*-value	AOR (95% CI)	*p*-value
Age				
Years	1.025 (1.000–1.050)	0.048	0.994 (0.959–1.030)	0.738
Duration of antihypertensive medication use				
Years	1.122 (1.056–1.193)	0.000	1.080 (0.996–1.170)	0.062
Number of antihypertensive medications used	1.478 (0.976–2.239)	0.065	1.496 (0.804–2.784)	0.204
Hydrochlorothiazide	0.659 (0.392–1.109)	0.116	0.837 (0.404–1.732)	0.632
Nifedipine	2.865 (1.502–5.465)	0.001	2.389 (0.900–6.340)	0.080
Other medications				
Yes	1.870 (1.090–3.207)	0.023	2.633 (0.598–11.587)	0.200
Number of other medications	1.262 (0.949–1.678)	0.109	0.876 (0.401–1.917)	0.741
Tiredness				
Yes	3.122 (1.825–5.348)	0.000	3.802 (1.723–8.391)	0.001
Sweats				
Yes	2.055 (1.023–4.128)	0.043	0.790 (0.266–2.342)	0.670
Nausea				
Yes	1.779 (0.843–3.756)	0.131	0.654 (0.190–2.260)	0.503
Palpitation				
Yes	1.995 (1.159–3.432)	0.013	2.014 (0.790–5.137)	0.143
Cold hands and feet				
Yes	3.098 (1.391–6.899)	0.006	1.676 (0.463–6.064)	0.431
Muscle pain				
Yes	2.971 (1.286–6.864)	0.011	5.199 (1.407–19.214)	0.013
Cramps				
Yes	1.918 (0.781–4.711)	0.155	3.073 (0.632–14.933)	0.164
Headache				
Yes	1.444 (0.860–2.424)	0.164	1.062 (0.454–2.488)	0.889
Anxiety				
Yes	1.909 (0.876–4.162)	0.104	0.906 (0.247–3.331)	0.882
Sleep poorly				
Yes	2.061 (1.054–4.029)	0.035	4.891 (1.578–15.160)	0.006
Skin rash				
Yes	3.171 (1.029–9.774)	0.044	2.132 (0.373–12.193)	0.395
Decreased sexual desire				
Yes	0.494 (0.229–1.065)	0.072	0.643 (0.203–2.043)	0.454
Number of adverse effects	1.140 (1.039–1.250)	0.005	0.776 (0.592–1.017)	0.066
Symptoms are caused by medications				
I don’t know	1		1	
Yes	1.348 (0.669–2.714)	0.403	3.249 (1.248–8.456)	0.016
No	2.141 (1.131–4.049)	0.019	1.720 (0.673–4.392)	0.257
Symptoms caused to change or stop medications	10.554 (4.169–26.717)	0.000	16.104 (4.164–62.290)	0.000
Regular exercise				
Yes	0.363 (0.209–0.632)	0.000	0.170 (0.072–0.402)	0.000

Binary logistic regression identified statistically significant associations between the antihypertensive medications and associated ADRs. Nausea, constipation, palpitation, swollen feet or legs, cold hands or feet, cramps, persistent dry cough, skin rash, frequent urination, and decreased sexual desires were associated with one or more of the following antihypertensive medications: furosemide, hydrochlorothiazide, amlodipine, nifedipine, atenolol, and enalapril (Table 5).

**Table 5:** Relationship between ADRs and specific antihypertensive medications

ADRs	Medications	COR	*p*-value
Nausea	Furosemide	5.509 (1.173–25.860)	0.031
Constipation	Furosemide	4.750 (1.017–22.186)	0.048
Palpitation	Amlodipine	2.136 (1.200–3.802)	0.010
	Furosemide	5.357 (1.017–28.231)	0.048
Swollen feet or legs	Amlodipine	2.263 (1.228–4.169)	0.009
	Nifedipine	2.224 (1.145–4.317)	0.018
	Atenolol	12.828 (1.406–17.055)	0.024
Cold hands or feet	Furosemide	6.231 (1.321–29.393)	0.021
	Atenolol	12.577 (2.008–78.782)	0.007
Cramps	Nifedipine	3.421 (1.352–8.653)	0.009
	Amlodipine	3.329 (1.435–7.723)	0.005
	Enalapril	2.533 (1.271–5.044)	0.008
Persistent dry cough	Amlodipine	0.448 (0.206–0.974)	0.043
	Enalapril	6.348 (3.140–12.831)	< 0.001
Skin rash	Furosemide	7.667 (1.346–43.658)	0.022
Frequent urination	Hydrochlorothiazide	6.866 (3.629–12.988)	< 0.001
	Nifedipine	0.428 (0.207–0.886)	0.022
Decreased sexual desire	Hydrochlorothiazide	4.325 (1.831–10.216)	0.001
	Enalapril	0.458 (0.221–0.950)	0.036

## Discussion

This study tried to see whether adverse effects due to antihypertensive medications influence the medication-taking behavior of hypertensive patients. Based on the findings of this study, the medication-taking behavior of the patients was negatively affected by antihypertensive medications adverse effects. In general, nearly a third of patients reported poor adherence. One-fifth of the study participants think that their symptoms were caused by their antihypertensive medications and another 13.4% of the study participants reported that, symptoms caused them to change their medication administration behavior.

Several other studies reported that medication-taking behavior can be affected by adverse effects [26, 28–35]. Grassi et al. reported that up to more than half of antihypertensive medication discontinuation can be attributed to adverse effects [26]. In another study, Grégoire et al. reported that patients who perceived adverse effects from their antihypertensive medications were 1.91 times more likely to discontinue their initial antihypertensive medications [31]. One Palestinian study also reported a 4.6 fold increase in the rate of poor adherence in patients who perceived adverse effects [24]. Even though none of the study participants in the current study discontinued their antihypertensive medications, a three-fold increase in the rates of non-adherence were observed.

In a systematic review of qualitative studies, tiredness, urinary frequency, ankle swelling, lethargy, and impotence were mentioned as troublesome adverse effects [33] while tiredness, muscle pain, and poor sleep were associated with poor adherence in the current study. On the other hand, patients who reported to have regular physical exercise were more likely to be adherent to their antihypertensive medications. Better knowledge about the disease and its management could contribute to better adherence by these patients.

Efforts that focus on improving the knowledge and awareness of patients is crucial for optimal treatment outcomes especially in patients with diseases such as hypertension. Hypertensive patients do not usually present with symptoms but can ultimately experience serious cardiovascular consequences. The fact that they don’t usually have symptoms coupled with the adverse effects can discourage them to be adherent to their antihypertensive medications [29, 32, 35].

Forgetfulness was the most commonly mentioned reason for poor adherence to antihypertensive medications. This has been reported in other studies [24, 28, 29, 32]. Fasting has been reported as the second most common cause of poor adherence. Therefore, depending on each patient’s case, clinicians should work closely with patients and select the appropriate regimen and time of administration in order to improve adherence.

HCT, enalapril, and amlodipine were the most commonly prescribed medications. While it is believed that there might be differences in the adverse effect profiles of different antihypertensive medications, no difference was observed in the level of adherence among patients different antihypertensive medications. On the other hand, hypertensive patients usually present with other chronic conditions for which they receive other medications. This increases the pill burden and complexity of regimens and has been attributed to poor adherence. In the current study, 34.5% patients used medications for the management of other chronic conditions. While this decreased the rate of adherence to antihypertensive medications upon univariate analysis, it failed to independently predict adherence upon multivariate analysis.

Just below half of the study participants (43.37%) were taking monotherapy while the remaining patients were on two or more combination antihypertensive medications. The complexity of medication regimens including the number of antihypertensive medications was associated with poor adherence [28, 29]. However, the number of medications was not found to be statistically significant in predicting adherence in the current study. Despite this, promoting the use of fixed-dose combination antihypertensive medications (FDC) may improve medication adherence. Several studies reported advantages of FDC in terms of reducing adverse effects and simplifying regimens and thereby improving adherence [36]. However, availability of these FDC is also another issue in the current set up. On the other hand, selecting antihypertensive medications with better safety profile can help improve medication adherence. Studies reported that Angiotensin II Receptor Blockers (ARBs) have similar, if not better, safety profile than other antihypertensive classes and can improve medication adherence in hypertensive patients [26, 29, 36]. None of the study participants in the current study received ARBs and the use of these medications might be hindered by availability and affordability issues. ACEIs are better tolerated than most other antihypertensive medications; hence, they could be preferably used in the absence of ARBs [26].

Adherence to antihypertensive medications should be stressed as it is associated with poor outcomes. It has been associated with the inability to achieve BP control [26] and a significant increase in the risk for all-cause mortality and hospitalization due to cardiovascular events (hazard ratio = 1.57) [7]. This necessitates efforts to improve medication adherence. One effort could be involving pharmacists in direct patient care as it has been demonstrated that pharmacist-led interventions improve antihypertensive medication adherence and BP control [37].

Interpretation of the findings of this study should be in light of the following limitations. This is a single-center cross-sectional study with small sample size and hence generalization to other settings should be done with caution. In addition, the study relied on patients reports of adverse reactions and may be subject to recall bias as it requires recalling missed doses in the past 3 months. It is also impossible to differentiate whether the adverse effects were due to the antihypertensive medications or other medications the patients were taking.

Statistically significant associations were identified among antihypertensive medications and a number of ADRs. As one might expect from its pharmacologic action, hydrochlorothiazide was associated with a six-fold increase in the number of patients experiencing frequent urination. As reported from clinical trials [38, 39], enalapril use was associated with cough. Other ADRs include cold hands or feet with furosemide and atenolol and swollen feet or legs with CCBs (amlodipine and nifedipine) and atenolol. In addition, furosemide was associated with nausea, constipation, palpitation, and skin rash.

## Conclusion

Adverse effect significantly contributes to antihypertensive medication non-adherence in hypertensive patients. Most patients don’t know if their symptoms were due to their antihypertensive medications. However, patients who thought that their symptoms were due to the antihypertensive medications they were taking tend to be non-adherent. Patients should be counseled about adverse effects common to their antihypertensive medications and should be directly involved in the decision process. Studies with prospective study designs should be done in the future to help better understand the association between adverse effects and non-adherence.

## Abbreviations

ACEIs: Angiotensin converting enzyme inhibitors
AOR: Adjusted odds ratio
ARB: Angiotensin II receptor blockers
BP: Blood pressure
CCBs: Calcium channel blockers
COR: Crude odds ratio
FDC: Fixed dose combination
SPSS: Statistical package for social science
UOGCSH: University of Gondar Comprehensive Specialized Hospital
